# Editorial: Gastrointestinal function and nutrition in pediatric critical care

**DOI:** 10.3389/fped.2022.1056544

**Published:** 2022-10-28

**Authors:** Haifa Mtaweh, Enid E. Martinez

**Affiliations:** ^1^ Department of Pediatrics, University of Toronto, Toronto, ON, Canada; ^2^ Staff Physician, Department of Critical Care Medicine, SickKids, Toronto, ON, Canada; ^3^ Department of Anaesthesia, Harvard Medical School, Boston, MA, United States; ^4^ Department of Anesthesiology, Critical Care and Pain Medicine, Boston Children's Hospital, Boston, MA, United States

**Keywords:** enteral nutrition, critical care, children, pediatric, gastrointestinal, congenital heart disease, ultrasound, feeding intolerance

**Editorial on the Research Topic** Gastrointestinal function and nutrition in pediatric critical care By Mtaweh H and Martinez EE. (2022) Front. Pediatr. 10: 1056544. doi: 10.3389/fped.2022.1056544

In this Research Topic Section for Frontiers in Pediatrics we have focused on gastrointestinal function and enteral nutrition primarily in critically ill children. Early and optimal provision of enteral nutrition has been associated with improved clinical outcomes in critically ill children (1). However, challenges associated with patient factors, such as underlying disease and gastrointestinal function, and practice factors, such as logistics of feeding tube placement, can often impede initiation and delivery of adequate enteral nutrition. In this Research Topic, we present a collection of original research work and reviews on varied topics related to such patient and practice factors. These studies reflect a continued impetus for improving our practices surrounding enteral nutrition for critically ill children and have the potential to influence both clinical practice and future areas of research.

Enteral nutrition in critically ill patients remains the standard of care to hasten recovery and reduce iatrogenic complications (2–5). This delivery is however limited by the underlying illness phenotype and the gastrointestinal function of patients such as is the case with patients with congenital heart disease (CHD) or necrotizing enterocolitis (NEC) (6–10). In this Research Topic, we included studies that presented alternatives to improving enteral nutrition in such patient populations. Zhang and colleagues demonstrate the utility of standardized nutritional intake regimens in children with unrestricted ventricular septal defects in improving preoperative state and recovery. Ni and colleagues demonstrate the feasibility of high caloric density enteral intake in achieving caloric and protein goals with improved short term post-operative outcomes without increasing the risk of gastrointestinal intolerance. Song and colleagues constructed a nomogram to allow the early identification of different NEC stages, which would facilitate early therapy including nutrition. Lastly, the relationship between physical rehabilitation, nutritional therapy, and recovery from critical illness is physiologically valid as is shown in recent studies (11–13). Yu and colleagues studied the relation between mobility, particularly cycling, and gastrointestinal symptoms that are commonly reported in critical illness and could hinder enteral nutrition delivery.

Providing optimal enteral nutrition is also limited by challenges associated with nutrition practices at the bedside (13, 14). Feeding interruptions is a commonly reported cause for inadequate enteral nutrition delivery (13, 14). Nabialek and colleagues show in a world-wide survey that nil per os durations prior to performing planned extubations and time to initiate nutrition after extubation vary widely. Enteral nutrition intolerance is another common cause for feeding interruptions, and the most commonly reported sign and symptom to determine intolerance is the measurement of gastric residual volume (GRV) (14). The measurement of GRV, however, has been shown in observational studies to not be associated consistently with delayed gastric emptying and may limit optimal enteral nutrition delivery (15). In this Research Topic, Valla and colleagues have also shown the lack of accuracy from GRV measurements by correlating gastric volume by GRV measurement with gastric volume as measured by point of care gastric ultrasound. This work also highlights the growing use of point of care gastric ultrasound in pediatric critical care practice. In a scoping review, Valla and colleagues expand on the multiple uses of the point of care gastric ultrasound, which is on the rise and includes measurement of gastric contents, gastric volume, gastric emptying and enteric tube placement. A common alternative approach to overcoming enteral nutrition intolerance and reducing feeding interruptions is the use of post-pyloric feeding route. Utilization of post-pyloric tubes varies widely and when attempts at placement are unsuccessful it can result in delayed initiation of enteral nutrition. In a multi-center, prospective study, Xu and colleagues showed that a two-stage approach for post-pyloric tube placement, first with self-migrating tubes and prokinetics, and then with blind guidance by experienced operators resulted in a total 91.1% success rate in tube placement. Once enteral nutrition has been initiated, many critically ill patients are at risk for refeeding syndrome. In a single-center retrospective study, Blanc and colleagues reported that 7.4% of patients had probable refeeding syndrome based on a greater than 10% change in electrolytes in the setting of starting nutrition, and of these patients 58.1% were considered to have severe refeeding syndrome.

This research topic addresses some of the highlighted domains in the latest international collaboration that summarized topics of priority in pediatric critical care nutrition research (14). This collection of work has added to the evidence in three of those domains: effective and safe delivery of enteral nutrition, assessment and management of enteral feeding intolerance, and nutrition therapies for specific populations. The evidence continues to emphasize the importance of enteral nutrition delivery in improving acute critical care outcomes and explored methods to counter the delay in initiating and achieving full nutritional prescription.
